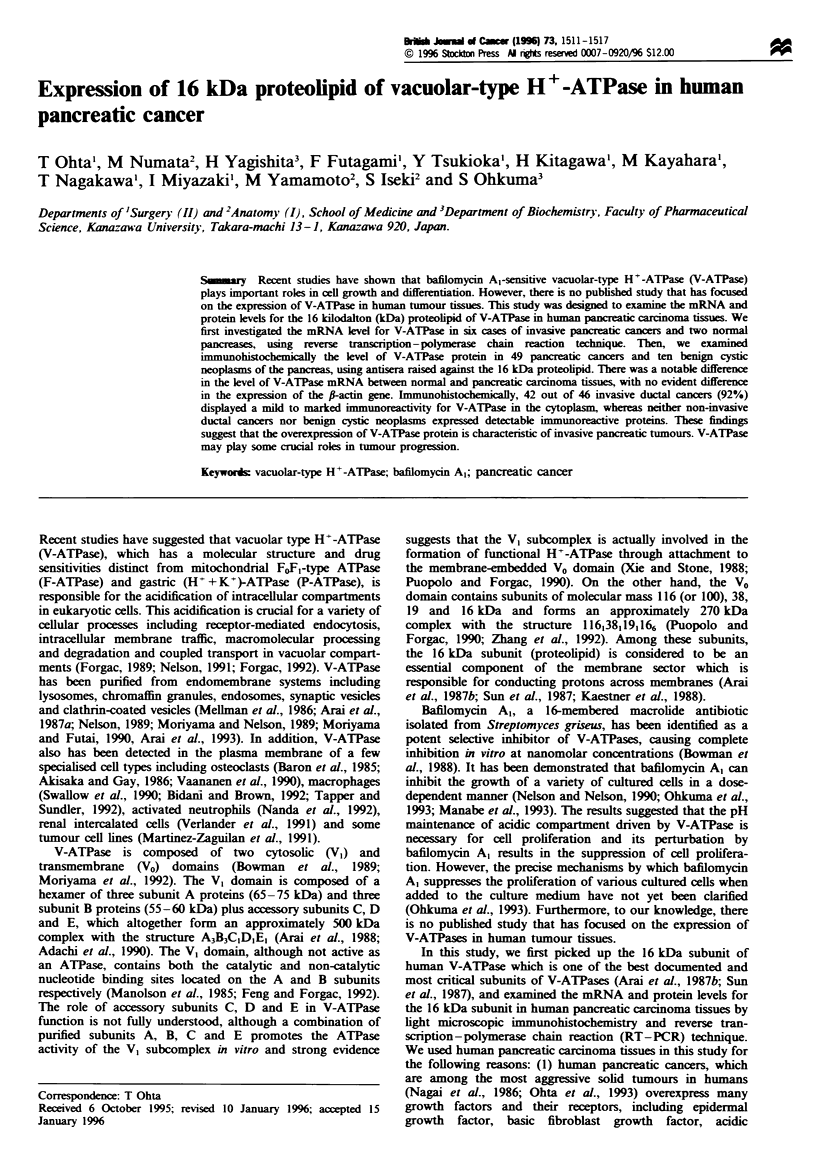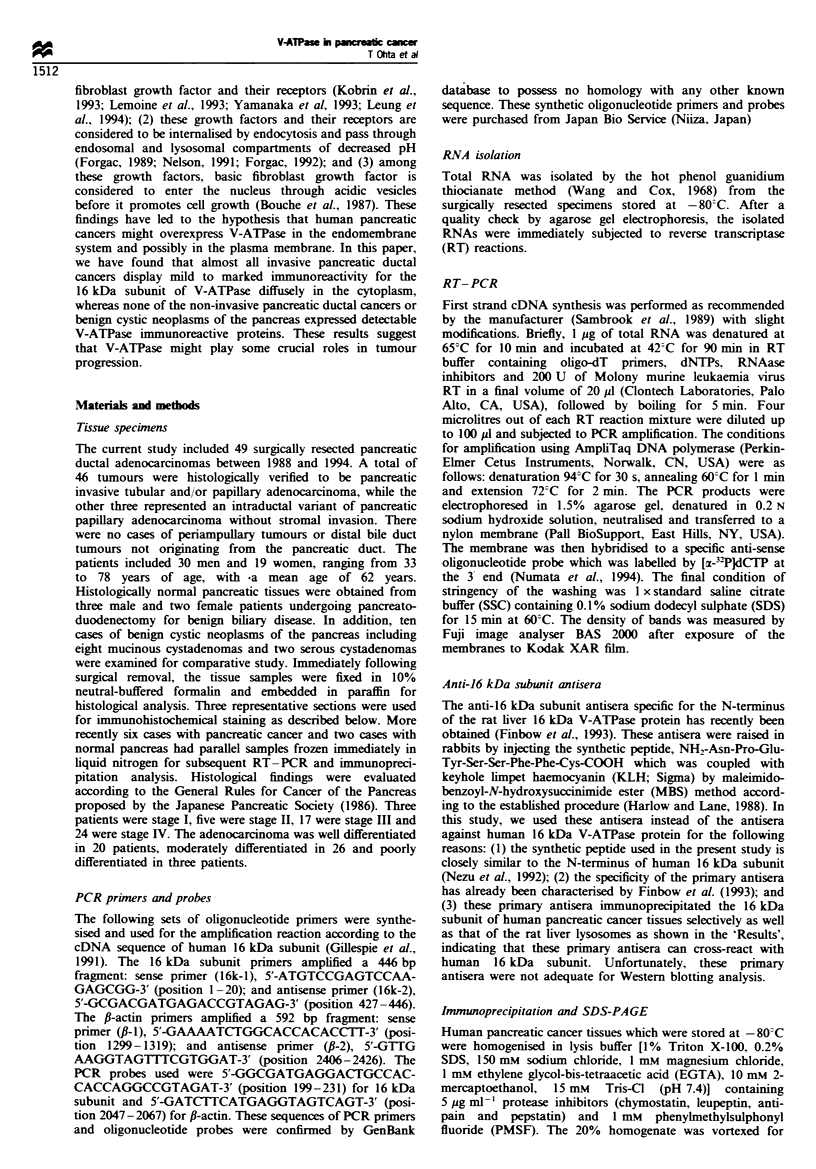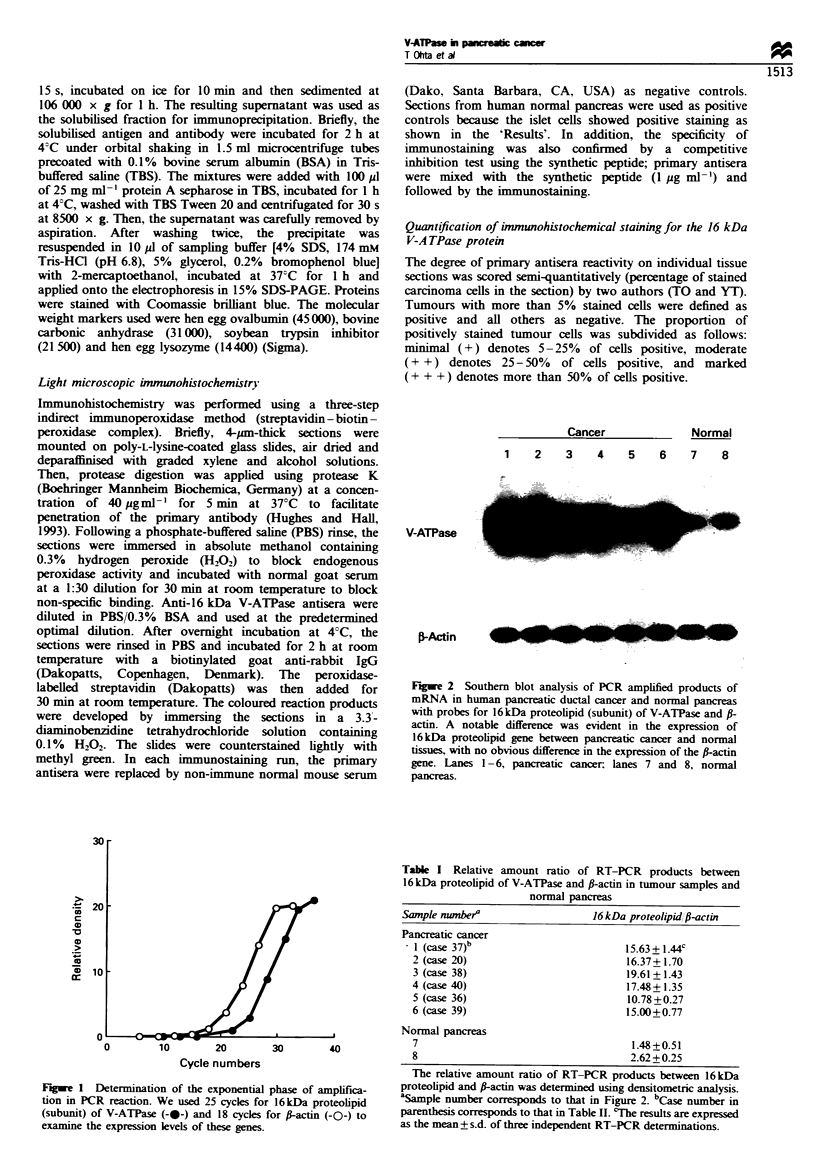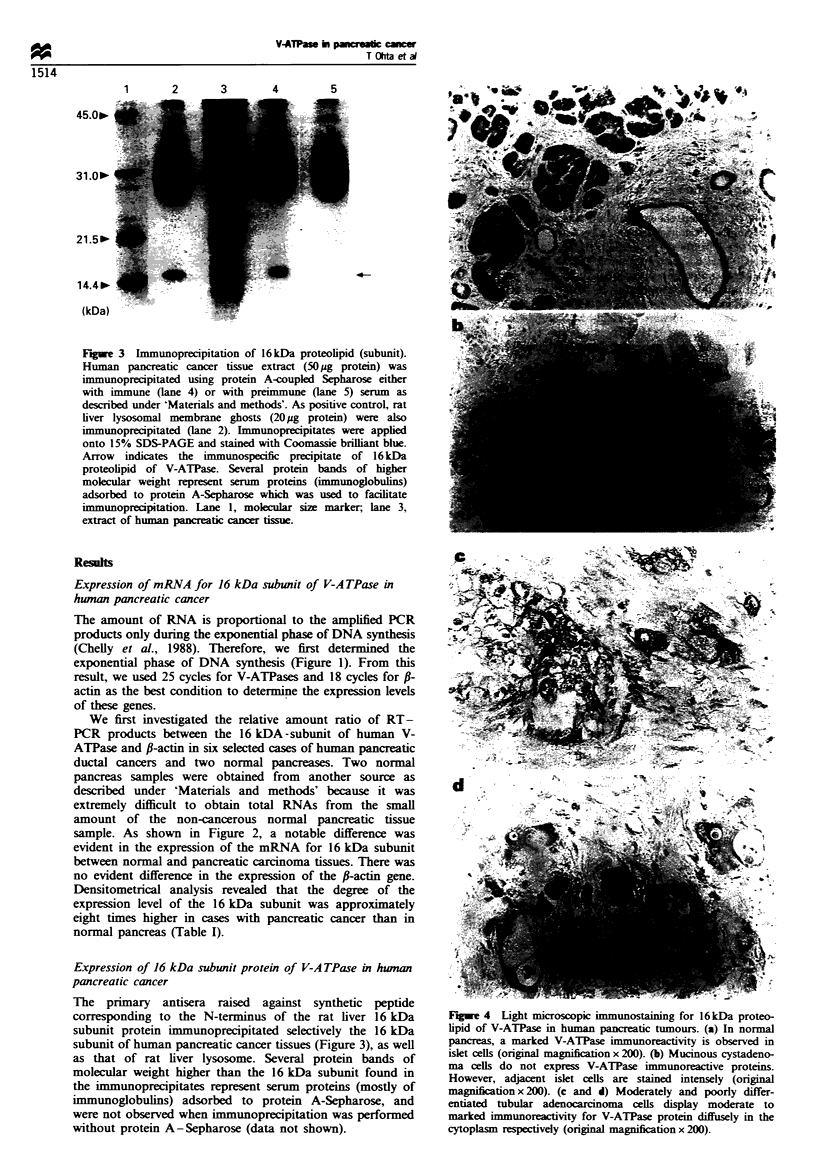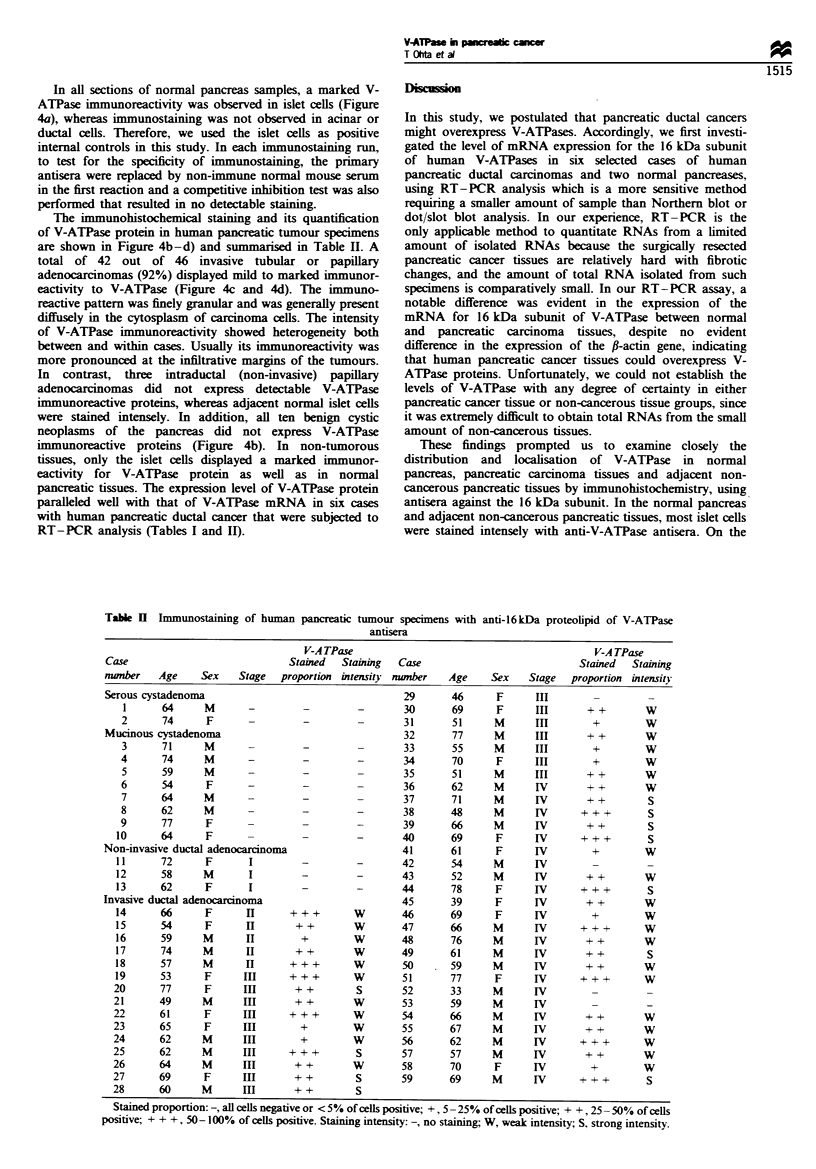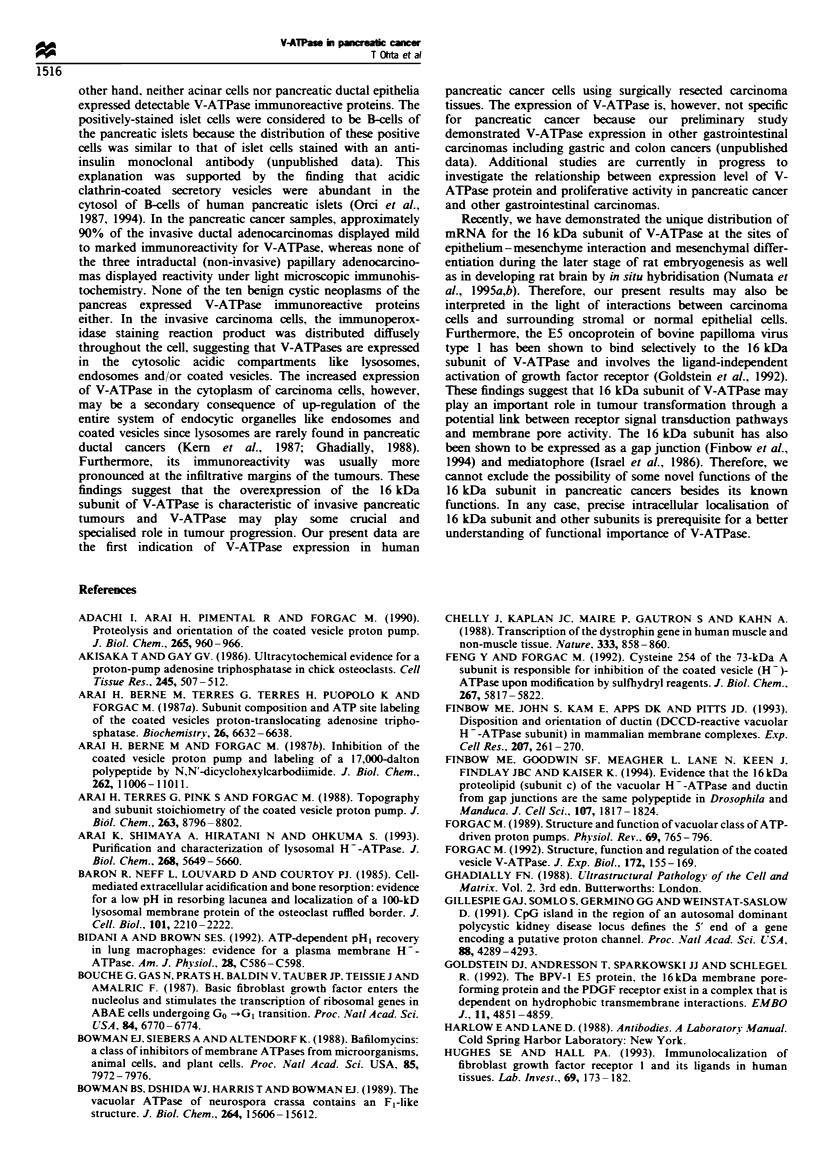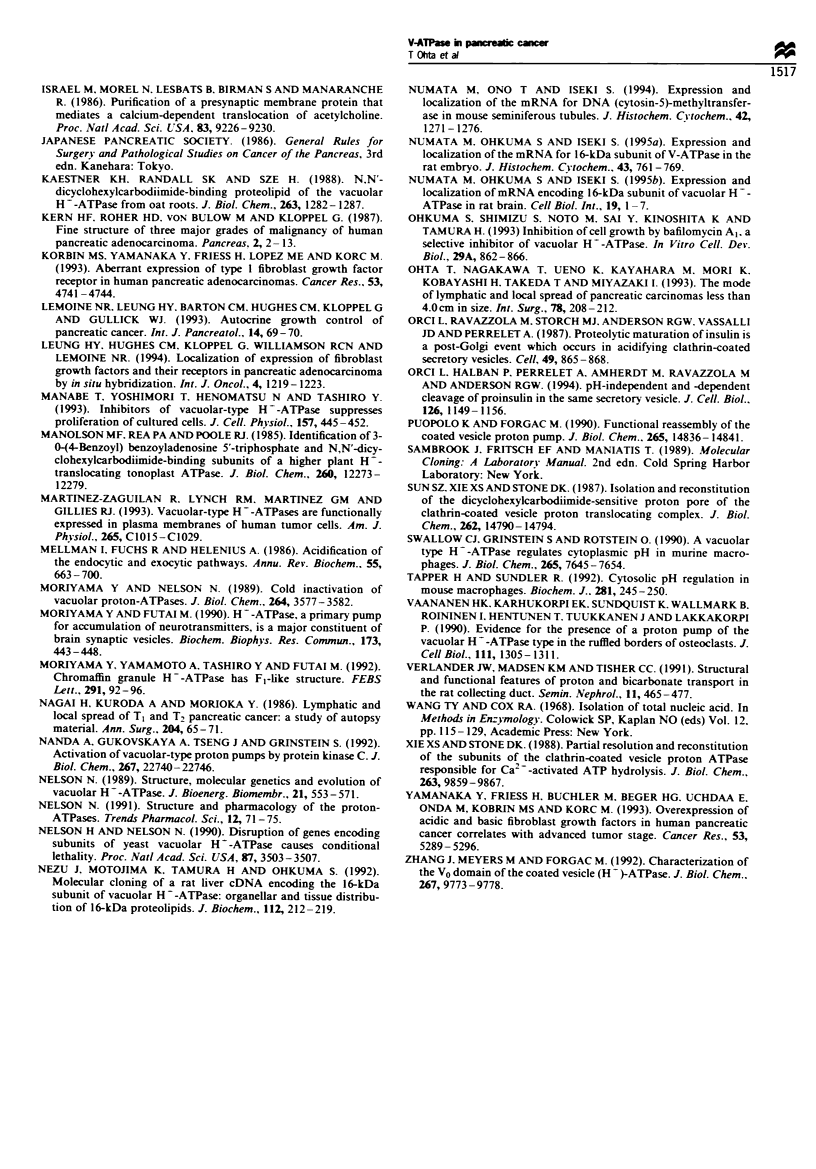# Expression of 16 kDa proteolipid of vacuolar-type H(+)-ATPase in human pancreatic cancer.

**DOI:** 10.1038/bjc.1996.285

**Published:** 1996-06

**Authors:** T. Ohta, M. Numata, H. Yagishita, F. Futagami, Y. Tsukioka, H. Kitagawa, M. Kayahara, T. Nagakawa, I. Miyazaki, M. Yamamoto, S. Iseki, S. Ohkuma

**Affiliations:** Department of Surgery (II), School of Medicine, Kanazawa University, Japan.

## Abstract

**Images:**


© 1996 Stockton Press All rights reserved 0007–0920/96 $12.00

### Expression of 16 kDa proteolipid of vacuolar-type $H^+$-ATPase in human pancreatic cancer

T Ohta[1], M Numata[2], H Yagishita[3], F Futagami[1], Y Tsukioka[1], H Kitagawa[1], M Kayahara[1], T Nagakawa[1], I Miyazaki[1], M Yamamoto[2], S Iseki[2] and S Ohkuma[3]

*Departments of [1]Surgery (II) and [2]Anatomy (I), School of Medicine and [3]Department of Biochemistry, Faculty of Pharmaceutical Science, Kanazawa University, Takara-machi 13–1, Kanazawa 920, Japan.*

**Summary** Recent studies have shown that bafilomycin $A_1$-sensitive vacuolar-type $H^+$-ATPase (V-ATPase) plays important roles in cell growth and differentiation. However, there is no published study that has focused on the expression of V-ATPase in human tumour tissues. This study was designed to examine the mRNA and protein levels for the 16 kilodalton (kDa) proteolipid of V-ATPase in human pancreatic carcinoma tissues. We first investigated the mRNA level for V-ATPase in six cases of invasive pancreatic cancers and two normal pancreases, using reverse transcription–polymerase chain reaction technique. Then, we examined immunohistochemically the level of V-ATPase protein in 49 pancreatic cancers and ten benign cystic neoplasms of the pancreas, using antisera raised against the 16 kDa proteolipid. There was a notable difference in the level of V-ATPase mRNA between normal and pancreatic carcinoma tissues, with no evident difference in the expression of the $\beta$-actin gene. Immunohistochemically, 42 out of 46 invasive ductal cancers (92%) displayed a mild to marked immunoreactivity for V-ATPase in the cytoplasm, whereas neither non-invasive ductal cancers nor benign cystic neoplasms expressed detectable immunoreactive proteins. These findings suggest that the overexpression of V-ATPase protein is characteristic of invasive pancreatic tumours. V-ATPase may play some crucial roles in tumour progression.

**Keywords:** vacuolar-type $H^+$-ATPase; bafilomycin $A_1$; pancreatic cancer

Recent studies have suggested that vacuolar type $H^+$-ATPase (V-ATPase), which has a molecular structure and drug sensitivities distinct from mitochondrial $F_0F_1$-type ATPase (F-ATPase) and gastric $(H^+ + K^+)$-ATPase (P-ATPase), is responsible for the acidification of intracellular compartments in eukaryotic cells. This acidification is crucial for a variety of cellular processes including receptor-mediated endocytosis, intracellular membrane traffic, macromolecular processing and degradation and coupled transport in vacuolar compartments (Forgac, 1989; Nelson, 1991; Forgac, 1992). V-ATPase has been purified from endomembrane systems including lysosomes, chromaffin granules, endosomes, synaptic vesicles and clathrin-coated vesicles (Mellman *et al.*, 1986; Arai *et al.*, 1987*a*; Nelson, 1989; Moriyama and Nelson, 1989; Moriyama and Futai, 1990, Arai *et al.*, 1993). In addition, V-ATPase also has been detected in the plasma membrane of a few specialised cell types including osteoclasts (Baron *et al.*, 1985; Akisaka and Gay, 1986; Vaananen *et al.*, 1990), macrophages (Swallow *et al.*, 1990; Bidani and Brown, 1992; Tapper and Sundler, 1992), activated neutrophils (Nanda *et al.*, 1992), renal intercalated cells (Verlander *et al.*, 1991) and some tumour cell lines (Martinez-Zaguilan *et al.*, 1991).

V-ATPase is composed of two cytosolic ($V_1$) and transmembrane ($V_0$) domains (Bowman *et al.*, 1989; Moriyama *et al.*, 1992). The $V_1$ domain is composed of a hexamer of three subunit A proteins (65–75 kDa) and three subunit B proteins (55–60 kDa) plus accessory subunits C, D and E, which altogether form an approximately 500 kDa complex with the structure $A_3B_3C_1D_1E_1$ (Arai *et al.*, 1988; Adachi *et al.*, 1990). The $V_1$ domain, although not active as an ATPase, contains both the catalytic and non-catalytic nucleotide binding sites located on the A and B subunits respectively (Manolson *et al.*, 1985; Feng and Forgac, 1992). The role of accessory subunits C, D and E in V-ATPase function is not fully understood, although a combination of purified subunits A, B, C and E promotes the ATPase activity of the $V_1$ subcomplex *in vitro* and strong evidence suggests that the $V_1$ subcomplex is actually involved in the formation of functional $H^+$-ATPase through attachment to the membrane-embedded $V_0$ domain (Xie and Stone, 1988; Puopolo and Forgac, 1990). On the other hand, the $V_0$ domain contains subunits of molecular mass 116 (or 100), 38, 19 and 16 kDa and forms an approximately 270 kDa complex with the structure $116_1 38_1 19_1 16_6$ (Puopolo and Forgac, 1990; Zhang *et al.*, 1992). Among these subunits, the 16 kDa subunit (proteolipid) is considered to be an essential component of the membrane sector which is responsible for conducting protons across membranes (Arai *et al.*, 1987*b*; Sun *et al.*, 1987; Kaestner *et al.*, 1988).

Bafilomycin $A_1$, a 16-membered macrolide antibiotic isolated from *Streptomyces griseus*, has been identified as a potent selective inhibitor of V-ATPases, causing complete inhibition *in vitro* at nanomolar concentrations (Bowman *et al.*, 1988). It has been demonstrated that bafilomycin $A_1$ can inhibit the growth of a variety of cultured cells in a dose-dependent manner (Nelson and Nelson, 1990; Ohkuma *et al.*, 1993; Manabe *et al.*, 1993). The results suggested that the pH maintenance of acidic compartment driven by V-ATPase is necessary for cell proliferation and its perturbation by bafilomycin $A_1$ results in the suppression of cell proliferation. However, the precise mechanisms by which bafilomycin $A_1$ suppresses the proliferation of various cultured cells when added to the culture medium have not yet been clarified (Ohkuma *et al.*, 1993). Furthermore, to our knowledge, there is no published study that has focused on the expression of V-ATPases in human tumour tissues.

In this study, we first picked up the 16 kDa subunit of human V-ATPase which is one of the best documented and most critical subunits of V-ATPases (Arai *et al.*, 1987*b*; Sun *et al.*, 1987), and examined the mRNA and protein levels for the 16 kDa subunit in human pancreatic carcinoma tissues by light microscopic immunohistochemistry and reverse transcription–polymerase chain reaction (RT–PCR) technique. We used human pancreatic carcinoma tissues in this study for the following reasons: (1) human pancreatic cancers, which are among the most aggressive solid tumours in humans (Nagai *et al.*, 1986; Ohta *et al.*, 1993) overexpress many growth factors and their receptors, including epidermal growth factor, basic fibroblast growth factor, acidic

Correspondence: T Ohta
Received 6 October 1995; revised 10 January 1996; accepted 15 January 1996

fibroblast growth factor and their receptors (Kobrin *et al.*, 1993; Lemoine *et al.*, 1993; Yamanaka *et al*, 1993; Leung *et al.*, 1994); (2) these growth factors and their receptors are considered to be internalised by endocytosis and pass through endosomal and lysosomal compartments of decreased pH (Forgac, 1989; Nelson, 1991; Forgac, 1992); and (3) among these growth factors, basic fibroblast growth factor is considered to enter the nucleus through acidic vesicles before it promotes cell growth (Bouche *et al.*, 1987). These findings have led to the hypothesis that human pancreatic cancers might overexpress V-ATPase in the endomembrane system and possibly in the plasma membrane. In this paper, we have found that almost all invasive pancreatic ductal cancers display mild to marked immunoreactivity for the 16 kDa subunit of V-ATPase diffusely in the cytoplasm, whereas none of the non-invasive pancreatic ductal cancers or benign cystic neoplasms of the pancreas expressed detectable V-ATPase immunoreactive proteins. These results suggest that V-ATPase might play some crucial roles in tumour progression.

### Materials and methods

### *Tissue specimens*

The current study included 49 surgically resected pancreatic ductal adenocarcinomas between 1988 and 1994. A total of 46 tumours were histologically verified to be pancreatic invasive tubular and/or papillary adenocarcinoma, while the other three represented an intraductal variant of pancreatic papillary adenocarcinoma without stromal invasion. There were no cases of periampullary tumours or distal bile duct tumours not originating from the pancreatic duct. The patients included 30 men and 19 women, ranging from 33 to 78 years of age, with a mean age of 62 years. Histologically normal pancreatic tissues were obtained from three male and two female patients undergoing pancreato-duodenectomy for benign biliary disease. In addition, ten cases of benign cystic neoplasms of the pancreas including eight mucinous cystadenomas and two serous cystadenomas were examined for comparative study. Immediately following surgical removal, the tissue samples were fixed in 10% neutral-buffered formalin and embedded in paraffin for histological analysis. Three representative sections were used for immunohistochemical staining as described below. More recently six cases with pancreatic cancer and two cases with normal pancreas had parallel samples frozen immediately in liquid nitrogen for subsequent RT-PCR and immunoprecipitation analysis. Histological findings were evaluated according to the General Rules for Cancer of the Pancreas proposed by the Japanese Pancreatic Society (1986). Three patients were stage I, five were stage II, 17 were stage III and 24 were stage IV. The adenocarcinoma was well differentiated in 20 patients, moderately differentiated in 26 and poorly differentiated in three patients.

### *PCR primers and probes*

The following sets of oligonucleotide primers were synthesised and used for the amplification reaction according to the cDNA sequence of human 16 kDa subunit (Gillespie *et al.*, 1991). The 16 kDa subunit primers amplified a 446 bp fragment: sense primer (16k-1), 5'-ATGTCCGAGTCCAA-GAGCGG-3' (position 1-20); and antisense primer (16k-2), 5'-GCGACGATGAGACCGTAGAG-3' (position 427-446). The $\beta$-actin primers amplified a 592 bp fragment: sense primer ($\beta$-1), 5'-GAAAATCTGGCACCACACCTT-3' (position 1299-1319); and antisense primer ($\beta$-2), 5'-GTTG AAGGTAGTTTCGTGGAT-3' (position 2406-2426). The PCR probes used were 5'-GGCGATGAGGACTGCCAC-CACCAGGCCGTAGAT-3' (position 199-231) for 16 kDa subunit and 5'-GATCTTCATGAGGTAGTCAGT-3' (position 2047-2067) for $\beta$-actin. These sequences of PCR primers and oligonucleotide probes were confirmed by GenBank database to possess no homology with any other known sequence. These synthetic oligonucleotide primers and probes were purchased from Japan Bio Service (Niiza, Japan)

### *RNA isolation*

Total RNA was isolated by the hot phenol guanidium thiocianate method (Wang and Cox, 1968) from the surgically resected specimens stored at $-80^{\circ}C$. After a quality check by agarose gel electrophoresis, the isolated RNAs were immediately subjected to reverse transcriptase (RT) reactions.

### *RT-PCR*

First strand cDNA synthesis was performed as recommended by the manufacturer (Sambrook *et al.*, 1989) with slight modifications. Briefly, 1 $\mu g$ of total RNA was denatured at 65°C for 10 min and incubated at 42°C for 90 min in RT buffer containing oligo-dT primers, dNTPs, RNAase inhibitors and 200 U of Molony murine leukaemia virus RT in a final volume of 20 $\mu l$ (Clontech Laboratories, Palo Alto, CA, USA), followed by boiling for 5 min. Four microlitres out of each RT reaction mixture were diluted up to 100 $\mu l$ and subjected to PCR amplification. The conditions for amplification using AmpliTaq DNA polymerase (Perkin-Elmer Cetus Instruments, Norwalk, CN, USA) were as follows: denaturation 94°C for 30 s, annealing 60°C for 1 min and extension 72°C for 2 min. The PCR products were electrophoresed in 1.5% agarose gel, denatured in 0.2 N sodium hydroxide solution, neutralised and transferred to a nylon membrane (Pall BioSupport, East Hills, NY, USA). The membrane was then hybridised to a specific anti-sense oligonucleotide probe which was labelled by [$\alpha$-$^{32}$P]dCTP at the 3' end (Numata *et al.*, 1994). The final condition of stringency of the washing was 1 × standard saline citrate buffer (SSC) containing 0.1% sodium dodecyl sulphate (SDS) for 15 min at 60°C. The density of bands was measured by Fuji image analyser BAS 2000 after exposure of the membranes to Kodak XAR film.

### *Anti-16 kDa subunit antisera*

The anti-16 kDa subunit antisera specific for the N-terminus of the rat liver 16 kDa V-ATPase protein has recently been obtained (Finbow *et al.*, 1993). These antisera were raised in rabbits by injecting the synthetic peptide, $NH_2$-Asn-Pro-Glu-Tyr-Ser-Ser-Phe-Phe-Cys-COOH which was coupled with keyhole limpet haemocyanin (KLH; Sigma) by maleimido-benzoyl-*N*-hydroxysuccinimide ester (MBS) method according to the established procedure (Harlow and Lane, 1988). In this study, we used these antisera instead of the antisera against human 16 kDa V-ATPase protein for the following reasons: (1) the synthetic peptide used in the present study is closely similar to the N-terminus of human 16 kDa subunit (Nezu *et al.*, 1992); (2) the specificity of the primary antisera has already been characterised by Finbow *et al.* (1993); and (3) these primary antisera immunoprecipitated the 16 kDa subunit of human pancreatic cancer tissues selectively as well as that of the rat liver lysosomes as shown in the 'Results', indicating that these primary antisera can cross-react with human 16 kDa subunit. Unfortunately, these primary antisera were not adequate for Western blotting analysis.

### *Immunoprecipitation and SDS-PAGE*

Human pancreatic cancer tissues which were stored at $-80^{\circ}C$ were homogenised in lysis buffer [1% Triton X-100, 0.2% SDS, 150 mM sodium chloride, 1 mM magnesium chloride, 1 mM ethylene glycol-bis-tetraacetic acid (EGTA), 10 mM 2-mercaptoethanol, 15 mM Tris-Cl (pH 7.4)] containing 5 $\mu g\ ml^{-1}$ protease inhibitors (chymostatin, leupeptin, antipain and pepstatin) and 1 mM phenylmethylsulphonyl fluoride (PMSF). The 20% homogenate was vortexed for

15 s, incubated on ice for 10 min and then sedimented at 106 000 × $g$ for 1 h. The resulting supernatant was used as the solubilised fraction for immunoprecipitation. Briefly, the solubilised antigen and antibody were incubated for 2 h at 4°C under orbital shaking in 1.5 ml microcentrifuge tubes precoated with 0.1% bovine serum albumin (BSA) in Tris-buffered saline (TBS). The mixtures were added with 100 $\mu$l of 25 mg $ml^{-1}$ protein A sepharose in TBS, incubated for 1 h at 4°C, washed with TBS Tween 20 and centrifugated for 30 s at 8500 × g. Then, the supernatant was carefully removed by aspiration. After washing twice, the precipitate was resuspended in 10 $\mu$l of sampling buffer [4% SDS, 174 mM Tris-HCl (pH 6.8), 5% glycerol, 0.2% bromophenol blue] with 2-mercaptoethanol, incubated at 37°C for 1 h and applied onto the electrophoresis in 15% SDS-PAGE. Proteins were stained with Coomassie brilliant blue. The molecular weight markers used were hen egg ovalbumin (45 000), bovine carbonic anhydrase (31 000), soybean trypsin inhibitor (21 500) and hen egg lysozyme (14 400) (Sigma).

*Light microscopic immunohistochemistry*

Immunohistochemistry was performed using a three-step indirect immunoperoxidase method (streptavidin–biotin–peroxidase complex). Briefly, 4-$\mu$m-thick sections were mounted on poly-L-lysine-coated glass slides, air dried and deparaffinised with graded xylene and alcohol solutions. Then, protease digestion was applied using protease K (Boehringer Mannheim Biochemica, Germany) at a concentration of 40 $\mu$g $ml^{-1}$ for 5 min at 37°C to facilitate penetration of the primary antibody (Hughes and Hall, 1993). Following a phosphate-buffered saline (PBS) rinse, the sections were immersed in absolute methanol containing 0.3% hydrogen peroxide ($H_2O_2$) to block endogenous peroxidase activity and incubated with normal goat serum at a 1:30 dilution for 30 min at room temperature to block non-specific binding. Anti-16 kDa V-ATPase antisera were diluted in PBS/0.3% BSA and used at the predetermined optimal dilution. After overnight incubation at 4°C, the sections were rinsed in PBS and incubated for 2 h at room temperature with a biotinylated goat anti-rabbit IgG (Dakopatts, Copenhagen, Denmark). The peroxidase-labelled streptavidin (Dakopatts) was then added for 30 min at room temperature. The coloured reaction products were developed by immersing the sections in a 3,3'-diaminobenzidine tetrahydrochloride solution containing 0.1% $H_2O_2$. The slides were counterstained lightly with methyl green. In each immunostaining run, the primary antisera were replaced by non-immune normal mouse serum (Dako, Santa Barbara, CA, USA) as negative controls. Sections from human normal pancreas were used as positive controls because the islet cells showed positive staining as shown in the 'Results'. In addition, the specificity of immunostaining was also confirmed by a competitive inhibition test using the synthetic peptide; primary antisera were mixed with the synthetic peptide (1 $\mu$g $ml^{-1}$) and followed by the immunostaining.

*Quantification of immunohistochemical staining for the 16 kDa V-ATPase protein*

The degree of primary antisera reactivity on individual tissue sections was scored semi-quantitatively (percentage of stained carcinoma cells in the section) by two authors (TO and YT). Tumours with more than 5% stained cells were defined as positive and all others as negative. The proportion of positively stained tumour cells was subdivided as follows: minimal (+) denotes 5–25% of cells positive, moderate (+ +) denotes 25–50% of cells positive, and marked (+ + +) denotes more than 50% of cells positive.

**Figure 2** Southern blot analysis of PCR amplified products of mRNA in human pancreatic ductal cancer and normal pancreas with probes for 16 kDa proteolipid (subunit) of V-ATPase and β-actin. A notable difference was evident in the expression of 16 kDa proteolipid gene between pancreatic cancer and normal tissues, with no obvious difference in the expression of the β-actin gene. Lanes 1–6, pancreatic cancer; lanes 7 and 8, normal pancreas.

**Figure 1** Determination of the exponential phase of amplification in PCR reaction. We used 25 cycles for 16 kDa proteolipid (subunit) of V-ATPase (-●-) and 18 cycles for β-actin (-○-) to examine the expression levels of these genes.

**Table I** Relative amount ratio of RT–PCR products between 16 kDa proteolipid of V-ATPase and β-actin in tumour samples and normal pancreas

| *Sample number*[a] | *16 kDa proteolipid/β-actin* |
|---|---|
| Pancreatic cancer | |
| 1 (case 37)[b] | 15.63 ± 1.44[c] |
| 2 (case 20) | 16.37 ± 1.70 |
| 3 (case 38) | 19.61 ± 1.43 |
| 4 (case 40) | 17.48 ± 1.35 |
| 5 (case 36) | 10.78 ± 0.27 |
| 6 (case 39) | 15.00 ± 0.77 |
| Normal pancreas | |
| 7 | 1.48 ± 0.51 |
| 8 | 2.62 ± 0.25 |

The relative amount ratio of RT–PCR products between 16 kDa proteolipid and β-actin was determined using densitometric analysis. [a]Sample number corresponds to that in Figure 2. [b]Case number in parenthesis corresponds to that in Table II. [c]The results are expressed as the mean ± s.d. of three independent RT–PCR determinations.

**Figure 3** Immunoprecipitation of 16 kDa proteolipid (subunit). Human pancreatic cancer tissue extract (50 μg protein) was immunoprecipitated using protein A-coupled Sepharose either with immune (lane 4) or with preimmune (lane 5) serum as described under 'Materials and methods'. As positive control, rat liver lysosomal membrane ghosts (20 μg protein) were also immunoprecipitated (lane 2). Immunoprecipitates were applied onto 15% SDS-PAGE and stained with Coomassie brilliant blue. Arrow indicates the immunospecific precipitate of 16 kDa proteolipid of V-ATPase. Several protein bands of higher molecular weight represent serum proteins (immunoglobulins) adsorbed to protein A-Sepharose which was used to facilitate immunoprecipitation. Lane 1, molecular size marker; lane 3, extract of human pancreatic cancer tissue.

### Results

### *Expression of mRNA for 16 kDa subunit of V-ATPase in human pancreatic cancer*

The amount of RNA is proportional to the amplified PCR products only during the exponential phase of DNA synthesis (Chelly *et al.*, 1988). Therefore, we first determined the exponential phase of DNA synthesis (Figure 1). From this result, we used 25 cycles for V-ATPases and 18 cycles for β-actin as the best condition to determine the expression levels of these genes.

We first investigated the relative amount ratio of RT-PCR products between the 16 kDA-subunit of human V-ATPase and β-actin in six selected cases of human pancreatic ductal cancers and two normal pancreases. Two normal pancreas samples were obtained from another source as described under 'Materials and methods' because it was extremely difficult to obtain total RNAs from the small amount of the non-cancerous normal pancreatic tissue sample. As shown in Figure 2, a notable difference was evident in the expression of the mRNA for 16 kDa subunit between normal and pancreatic carcinoma tissues. There was no evident difference in the expression of the β-actin gene. Densitometrical analysis revealed that the degree of the expression level of the 16 kDa subunit was approximately eight times higher in cases with pancreatic cancer than in normal pancreas (Table I).

### *Expression of 16 kDa subunit protein of V-ATPase in human pancreatic cancer*

The primary antisera raised against synthetic peptide corresponding to the N-terminus of the rat liver 16 kDa subunit protein immunoprecipitated selectively the 16 kDa subunit of human pancreatic cancer tissues (Figure 3), as well as that of rat liver lysosome. Several protein bands of molecular weight higher than the 16 kDa subunit found in the immunoprecipitates represent serum proteins (mostly of immunoglobulins) adsorbed to protein A-Sepharose, and were not observed when immunoprecipitation was performed without protein A-Sepharose (data not shown).

**Figure 4** Light microscopic immunostaining for 16 kDa proteolipid of V-ATPase in human pancreatic tumours. (**a**) In normal pancreas, a marked V-ATPase immunoreactivity is observed in islet cells (original magnification × 200). (**b**) Mucinous cystadenoma cells do not express V-ATPase immunoreactive proteins. However, adjacent islet cells are stained intensely (original magnification × 200). (**c** and **d**) Moderately and poorly differentiated tubular adenocarcinoma cells display moderate to marked immunoreactivity for V-ATPase protein diffusely in the cytoplasm respectively (original magnification × 200).

In all sections of normal pancreas samples, a marked V-ATPase immunoreactivity was observed in islet cells (Figure 4a), whereas immunostaining was not observed in acinar or ductal cells. Therefore, we used the islet cells as positive internal controls in this study. In each immunostaining run, to test for the specificity of immunostaining, the primary antisera were replaced by non-immune normal mouse serum in the first reaction and a competitive inhibition test was also performed that resulted in no detectable staining.

The immunohistochemical staining and its quantification of V-ATPase protein in human pancreatic tumour specimens are shown in Figure 4b–d) and summarised in Table II. A total of 42 out of 46 invasive tubular or papillary adenocarcinomas (92%) displayed mild to marked immunoreactivity to V-ATPase (Figure 4c and 4d). The immunoreactive pattern was finely granular and was generally present diffusely in the cytosplasm of carcinoma cells. The intensity of V-ATPase immunoreactivity showed heterogeneity both between and within cases. Usually its immunoreactivity was more pronounced at the infiltrative margins of the tumours. In contrast, three intraductal (non-invasive) papillary adenocarcinomas did not express detectable V-ATPase immunoreactive proteins, whereas adjacent normal islet cells were stained intensely. In addition, all ten benign cystic neoplasms of the pancreas did not express V-ATPase immunoreactive proteins (Figure 4b). In non-tumorous tissues, only the islet cells displayed a marked immunoreactivity for V-ATPase protein as well as in normal pancreatic tissues. The expression level of V-ATPase protein paralleled well with that of V-ATPase mRNA in six cases with human pancreatic ductal cancer that were subjected to RT–PCR analysis (Tables I and II).

### Discussion

In this study, we postulated that pancreatic ductal cancers might overexpress V-ATPases. Accordingly, we first investigated the level of mRNA expression for the 16 kDa subunit of human V-ATPases in six selected cases of human pancreatic ductal carcinomas and two normal pancreases, using RT–PCR analysis which is a more sensitive method requiring a smaller amount of sample than Northern blot or dot/slot blot analysis. In our experience, RT–PCR is the only applicable method to quantitate RNAs from a limited amount of isolated RNAs because the surgically resected pancreatic cancer tissues are relatively hard with fibrotic changes, and the amount of total RNA isolated from such specimens is comparatively small. In our RT–PCR assay, a notable difference was evident in the expression of the mRNA for 16 kDa subunit of V-ATPase between normal and pancreatic carcinoma tissues, despite no evident difference in the expression of the $\beta$-actin gene, indicating that human pancreatic cancer tissues could overexpress V-ATPase proteins. Unfortunately, we could not establish the levels of V-ATPase with any degree of certainty in either pancreatic cancer tissue or non-cancerous tissue groups, since it was extremely difficult to obtain total RNAs from the small amount of non-cancerous tissues.

These findings prompted us to examine closely the distribution and localisation of V-ATPase in normal pancreas, pancreatic carcinoma tissues and adjacent non-cancerous pancreatic tissues by immunohistochemistry, using antisera against the 16 kDa subunit. In the normal pancreas and adjacent non-cancerous pancreatic tissues, most islet cells were stained intensely with anti-V-ATPase antisera. On the

**Table II** Immunostaining of human pancreatic tumour specimens with anti-16 kDa proteolipid of V-ATPase antisera

| *Case number* | *Age* | *Sex* | *Stage* | *V-ATPase* *Stained proportion* | *V-ATPase* *Staining intensity* | *Case number* | *Age* | *Sex* | *Stage* | *V-ATPase* *Stained proportion* | *V-ATPase* *Staining intensity* |
|---|---|---|---|---|---|---|---|---|---|---|---|
| Serous cystadenoma | | | | | | 29 | 46 | F | III | – | – |
| 1 | 64 | M | – | – | – | 30 | 69 | F | III | + + | W |
| 2 | 74 | F | – | – | – | 31 | 51 | M | III | + | W |
| Mucinous cystadenoma | | | | | | 32 | 77 | M | III | + + | W |
| 3 | 71 | M | – | – | – | 33 | 55 | M | III | + | W |
| 4 | 74 | M | – | – | – | 34 | 70 | F | III | + | W |
| 5 | 59 | M | – | – | – | 35 | 51 | M | III | + + | W |
| 6 | 54 | F | – | – | – | 36 | 62 | M | IV | + + | W |
| 7 | 64 | M | – | – | – | 37 | 71 | M | IV | + + | S |
| 8 | 62 | M | – | – | – | 38 | 48 | M | IV | + + + | S |
| 9 | 77 | F | – | – | – | 39 | 66 | M | IV | + + | S |
| 10 | 64 | F | – | – | – | 40 | 69 | F | IV | + + + | S |
| Non-invasive ductal adenocarcinoma | | | | | | 41 | 61 | F | IV | + | W |
| 11 | 72 | F | I | – | – | 42 | 54 | M | IV | – | – |
| 12 | 58 | M | I | – | – | 43 | 52 | M | IV | + + | W |
| 13 | 62 | F | I | – | – | 44 | 78 | F | IV | + + + | S |
| Invasive ductal adenocarcinoma | | | | | | 45 | 39 | F | IV | + + | W |
| 14 | 66 | F | II | + + + | W | 46 | 69 | F | IV | + | W |
| 15 | 54 | F | II | + + | W | 47 | 66 | M | IV | + + + | W |
| 16 | 59 | M | II | + | W | 48 | 76 | M | IV | + + | W |
| 17 | 74 | M | II | + + | W | 49 | 61 | M | IV | + + | S |
| 18 | 57 | M | II | + + + | W | 50 | 59 | M | IV | + + | W |
| 19 | 53 | F | III | + + + | W | 51 | 77 | F | IV | + + + | W |
| 20 | 77 | F | III | + + | S | 52 | 33 | M | IV | – | – |
| 21 | 49 | M | III | + + | W | 53 | 59 | M | IV | – | – |
| 22 | 61 | F | III | + + + | W | 54 | 66 | M | IV | + + | W |
| 23 | 65 | F | III | + | W | 55 | 67 | M | IV | + + | W |
| 24 | 62 | M | III | + | W | 56 | 62 | M | IV | + + + | W |
| 25 | 62 | M | III | + + + | S | 57 | 57 | M | IV | + + | W |
| 26 | 64 | M | III | + + | W | 58 | 70 | F | IV | + | W |
| 27 | 69 | F | III | + + | S | 59 | 69 | M | IV | + + + | S |
| 28 | 60 | M | III | + + | S | | | | | | |

Stained proportion: –, all cells negative or < 5% of cells positive; +, 5–25% of cells positive; + +, 25–50% of cells positive; + + +, 50–100% of cells positive. Staining intensity: –, no staining; W, weak intensity; S, strong intensity.

other hand, neither acinar cells nor pancreatic ductal epithelia expressed detectable V-ATPase immunoreactive proteins. The positively-stained islet cells were considered to be B-cells of the pancreatic islets because the distribution of these positive cells was similar to that of islet cells stained with an anti-insulin monoclonal antibody (unpublished data). This explanation was supported by the finding that acidic clathrin-coated secretory vesicles were abundant in the cytosol of B-cells of human pancreatic islets (Orci *et al.*, 1987, 1994). In the pancreatic cancer samples, approximately 90% of the invasive ductal adenocarcinomas displayed mild to marked immunoreactivity for V-ATPase, whereas none of the three intraductal (non-invasive) papillary adenocarcinomas displayed reactivity under light microscopic immunohistochemistry. None of the ten benign cystic neoplasms of the pancreas expressed V-ATPase immunoreactive proteins either. In the invasive carcinoma cells, the immunoperoxidase staining reaction product was distributed diffusely throughout the cell, suggesting that V-ATPases are expressed in the cytosolic acidic compartments like lysosomes, endosomes and/or coated vesicles. The increased expression of V-ATPase in the cytoplasm of carcinoma cells, however, may be a secondary consequence of up-regulation of the entire system of endocytic organelles like endosomes and coated vesicles since lysosomes are rarely found in pancreatic ductal cancers (Kern *et al.*, 1987; Ghadially, 1988). Furthermore, its immunoreactivity was usually more pronounced at the infiltrative margins of the tumours. These findings suggest that the overexpression of the 16 kDa subunit of V-ATPase is characteristic of invasive pancreatic tumours and V-ATPase may play some crucial and specialised role in tumour progression. Our present data are the first indication of V-ATPase expression in human pancreatic cancer cells using surgically resected carcinoma tissues. The expression of V-ATPase is, however, not specific for pancreatic cancer because our preliminary study demonstrated V-ATPase expression in other gastrointestinal carcinomas including gastric and colon cancers (unpublished data). Additional studies are currently in progress to investigate the relationship between expression level of V-ATPase protein and proliferative activity in pancreatic cancer and other gastrointestinal carcinomas.

Recently, we have demonstrated the unique distribution of mRNA for the 16 kDa subunit of V-ATPase at the sites of epithelium–mesenchyme interaction and mesenchymal differentiation during the later stage of rat embryogenesis as well as in developing rat brain by *in situ* hybridisation (Numata *et al.*, 1995*a,b*). Therefore, our present results may also be interpreted in the light of interactions between carcinoma cells and surrounding stromal or normal epithelial cells. Furthermore, the E5 oncoprotein of bovine papilloma virus type 1 has been shown to bind selectively to the 16 kDa subunit of V-ATPase and involves the ligand-independent activation of growth factor receptor (Goldstein *et al.*, 1992). These findings suggest that 16 kDa subunit of V-ATPase may play an important role in tumour transformation through a potential link between receptor signal transduction pathways and membrane pore activity. The 16 kDa subunit has also been shown to be expressed as a gap junction (Finbow *et al.*, 1994) and mediatophore (Israel *et al.*, 1986). Therefore, we cannot exclude the possibility of some novel functions of the 16 kDa subunit in pancreatic cancers besides its known functions. In any case, precise intracellular localisation of 16 kDa subunit and other subunits is prerequisite for a better understanding of functional importance of V-ATPase.

### References

ADACHI I, ARAI H, PIMENTAL R AND FORGAC M. (1990). Proteolysis and orientation of the coated vesicle proton pump. *J. Biol. Chem.*, **265**, 960–966.

AKISAKA T AND GAY GV. (1986). Ultracytochemical evidence for a proton-pump adenosine triphosphatase in chick osteoclasts. *Cell Tissue Res.*, **245**, 507–512.

ARAI H, BERNE M, TERRES G, TERRES H, PUOPOLO K AND FORGAC M. (1987*a*). Subunit composition and ATP site labeling of the coated vesicles proton-translocating adenosine triphosphatase. *Biochemistry*, **26**, 6632–6638.

ARAI H, BERNE M AND FORGAC M. (1987*b*). Inhibition of the coated vesicle proton pump and labeling of a 17,000-dalton polypeptide by N,N′-dicyclohexylcarbodiimide. *J. Biol. Chem.*, **262**, 11006–11011.

ARAI H, TERRES G, PINK S AND FORGAC M. (1988). Topography and subunit stoichiometry of the coated vesicle proton pump. *J. Biôl. Chem.*, **263**, 8796–8802.

ARAI K, SHIMAYA A, HIRATANI N AND OHKUMA S. (1993). Purification and characterization of lysosomal $H^+$-ATPase. *J. Biol. Chem.*, **268**, 5649–5660.

BARON R, NEFF L, LOUVARD D AND COURTOY PJ. (1985). Cell-mediated extracellular acidification and bone resorption: evidence for a low pH in resorbing lacunea and localization of a 100-kD lysosomal membrane protein of the osteoclast ruffled border. *J. Cell. Biol.*, **101**, 2210–2222.

BIDANI A AND BROWN SES. (1992). ATP-dependent $pH_1$ recovery in lung macrophages: evidence for a plasma membrane $H^+$-ATPase. *Am. J. Physiol.*, **28**, C586–C598.

BOUCHE G, GAS N, PRATS H, BALDIN V, TAUBER JP, TEISSIE J AND AMALRIC F. (1987). Basic fibroblast growth factor enters the nucleolus and stimulates the transcription of ribosomal genes in ABAE cells undergoing $G_0 \rightarrow G_1$ transition. *Proc. Natl Acad. Sci. USA*, **84**, 6770–6774.

BOWMAN EJ, SIEBERS A AND ALTENDORF K. (1988). Bafilomycins: a class of inhibitors of membrane ATPases from microorganisms, animal cells, and plant cells. *Proc. Natl Acad. Sci.* USA, **85**, 7972–7976.

BOWMAN BS, DSHIDA WJ, HARRIS T AND BOWMAN EJ. (1989). The vacuolar ATPase of neurospora crassa contains an $F_1$-like structure. *J. Biol. Chem.*, **264**, 15606–15612.

CHELLY J, KAPLAN JC, MAIRE P, GAUTRON S AND KAHN A. (1988). Transcription of the dystrophin gene in human muscle and non-muscle tissue. *Nature*, **333**, 858–860.

FENG Y AND FORGAC M. (1992). Cysteine 254 of the 73-kDa A subunit is responsible for inhibition of the coated vesicle ($H^+$)-ATPase upon modification by sulfhydryl reagents. *J. Biol. Chem.*, **267**, 5817–5822.

FINBOW ME, JOHN S, KAM E, APPS DK AND PITTS JD. (1993). Disposition and orientation of ductin (DCCD-reactive vacuolar $H^+$-ATPase subunit) in mammalian membrane complexes. *Exp. Cell Res.*, **207**, 261–270.

FINBOW ME, GOODWIN SF, MEAGHER L, LANE N, KEEN J, FINDLAY JBC AND KAISER K. (1994). Evidence that the 16 kDa proteolipid (subunit c) of the vacuolar $H^+$-ATPase and ductin from gap junctions are the same polypeptide in *Drosophila* and *Manduca*. *J. Cell Sci.*, **107**, 1817–1824.

FORGAC M. (1989). Structure and function of vacuolar class of ATP-driven proton pumps. *Physiol. Rev.*, **69**, 765–796.

FORGAC M. (1992). Structure, function and regulation of the coated vesicle V-ATPase. *J. Exp. Biol.*, **172**, 155–169.

GHADIALLY FN. (1988). *Ultrastructural Pathology of the Cell and Matrix*. Vol. 2. 3rd edn. Butterworths: London.

GILLESPIE GAJ, SOMLO S, GERMINO GG AND WEINSTAT-SASLOW D. (1991). CpG island in the region of an autosomal dominant polycystic kidney disease locus defines the 5′ end of a gene encoding a putative proton channel. *Proc. Natl Acad. Sci. USA*, **88**, 4289–4293.

GOLDSTEIN DJ, ANDRESSON T, SPARKOWSKI JJ AND SCHLEGEL R. (1992). The BPV-1 E5 protein, the 16 kDa membrane pore-forming protein and the PDGF receptor exist in a complex that is dependent on hydrophobic transmembrane interactions. *EMBO J.*, **11**, 4851–4859.

HARLOW E AND LANE D. (1988). *Antibodies. A Laboratory Manual*. Cold Spring Harbor Laboratory: New York.

HUGHES SE AND HALL PA. (1993). Immunolocalization of fibroblast growth factor receptor 1 and its ligands in human tissues. *Lab. Invest.*, **69**, 173–182.

ISRAEL M, MOREL N, LESBATS B, BIRMAN S AND MANARANCHE R. (1986). Purification of a presynaptic membrane protein that mediates a calcium-dependent translocation of acetylcholine. *Proc. Natl Acad. Sci. USA*, **83**, 9226–9230.

JAPANESE PANCREATIC SOCIETY. (1986). *General Rules for Surgery and Pathological Studies on Cancer of the Pancreas*, 3rd edn. Kanehara: Tokyo.

KAESTNER KH, RANDALL SK AND SZE H. (1988). N,N'-dicyclohexylcarbodiimide-binding proteolipid of the vacuolar $H^+$-ATPase from oat roots. *J. Biol. Chem.*, **263**, 1282–1287.

KERN HF, ROHER HD, VON BULOW M AND KLOPPEL G. (1987). Fine structure of three major grades of malignancy of human pancreatic adenocarcinoma. *Pancreas*, **2**, 2–13.

KORBIN MS, YAMANAKA Y, FRIESS H, LOPEZ ME AND KORC M. (1993). Aberrant expression of type 1 fibroblast growth factor receptor in human pancreatic adenocarcinomas. *Cancer Res.*, **53**, 4741–4744.

LEMOINE NR, LEUNG HY, BARTON CM, HUGHES CM, KLOPPEL G AND GULLICK WJ. (1993). Autocrine growth control of pancreatic cancer. *Int. J. Pancreatol.*, **14**, 69–70.

LEUNG HY, HUGHES CM, KLOPPEL G, WILLIAMSON RCN AND LEMOINE NR. (1994). Localization of expression of fibroblast growth factors and their receptors in pancreatic adenocarcinoma by *in situ* hybridization. *Int. J. Oncol.*, **4**, 1219–1223.

MANABE T, YOSHIMORI T, HENOMATSU N AND TASHIRO Y. (1993). Inhibitors of vacuolar-type $H^+$-ATPase suppresses proliferation of cultured cells. *J. Cell. Physiol.*, **157**, 445–452.

MANOLSON MF, REA PA AND POOLE RJ. (1985). Identification of 3-0-(4-Benzoyl) benzoyladenosine 5'-triphosphate and N,N'-dicyclohexylcarbodiimide-binding subunits of a higher plant $H^+$-translocating tonoplast ATPase. *J. Biol. Chem.*, **260**, 12273–12279.

MARTINEZ-ZAGUILAN R, LYNCH RM, MARTINEZ GM AND GILLIES RJ. (1993). Vacuolar-type $H^+$-ATPases are functionally expressed in plasma membranes of human tumor cells. *Am. J. Physiol.*, **265**, C1015–C1029.

MELLMAN I, FUCHS R AND HELENIUS A. (1986). Acidification of the endocytic and exocytic pathways. *Annu. Rev. Biochem.*, **55**, 663–700.

MORIYAMA Y AND NELSON N. (1989). Cold inactivation of vacuolar proton-ATPases. *J. Biol. Chem.*, **264**, 3577–3582.

MORIYAMA Y AND FUTAI M. (1990). $H^+$-ATPase, a primary pump for accumulation of neurotransmitters, is a major constituent of brain synaptic vesicles. *Biochem. Biophys. Res. Commun.*, **173**, 443–448.

MORIYAMA Y, YAMAMOTO A, TASHIRO Y AND FUTAI M. (1992). Chromaffin granule $H^+$-ATPase has $F_1$-like structure. *FEBS Lett.*, **291**, 92–96.

NAGAI H, KURODA A AND MORIOKA Y. (1986). Lymphatic and local spread of $T_1$ and $T_2$ pancreatic cancer: a study of autopsy material. *Ann. Surg.*, **204**, 65–71.

NANDA A, GUKOVSKAYA A, TSENG J AND GRINSTEIN S. (1992). Activation of vacuolar-type proton pumps by protein kinase C. *J. Biol. Chem.*, **267**, 22740–22746.

NELSON N. (1989). Structure, molecular genetics and evolution of vacuolar $H^+$-ATPase. *J. Bioenerg. Biomembr.*, **21**, 553–571.

NELSON N. (1991). Structure and pharmacology of the proton-ATPases. *Trends Pharmacol. Sci.*, **12**, 71–75.

NELSON H AND NELSON N. (1990). Disruption of genes encoding subunits of yeast vacuolar $H^+$-ATPase causes conditional lethality. *Proc. Natl Acad. Sci. USA*, **87**, 3503–3507.

NEZU J, MOTOJIMA K, TAMURA H AND OHKUMA S. (1992). Molecular cloning of a rat liver cDNA encoding the 16-kDa subunit of vacuolar $H^+$-ATPase: organellar and tissue distribution of 16-kDa proteolipids. *J. Biochem.*, **112**, 212–219.

NUMATA M, ONO T AND ISEKI S. (1994). Expression and localization of the mRNA for DNA (cytosin-5)-methyltransferase in mouse seminiferous tubules. *J. Histochem. Cytochem.*, **42**, 1271–1276.

NUMATA M, OHKUMA S AND ISEKI S. (1995*a*). Expression and localization of the mRNA for 16-kDa subunit of V-ATPase in the rat embryo. *J. Histochem. Cytochem.*, **43**, 761–769.

NUMATA M, OHKUMA S AND ISEKI S. (1995*b*). Expression and localization of mRNA encoding 16-kDa subunit of vacuolar $H^+$-ATPase in rat brain. *Cell Biol. Int.*, **19**, 1–7.

OHKUMA S, SHIMIZU S, NOTO M, SAI Y, KINOSHITA K AND TAMURA H. (1993) Inhibition of cell growth by bafilomycin $A_1$, a selective inhibitor of vacuolar $H^+$-ATPase. *In Vitro Cell. Dev. Biol.*, **29A**, 862–866.

OHTA T, NAGAKAWA T, UENO K, KAYAHARA M, MORI K, KOBAYASHI H, TAKEDA T AND MIYAZAKI I. (1993). The mode of lymphatic and local spread of pancreatic carcinomas less than 4.0 cm in size. *Int. Surg.*, **78**, 208–212.

ORCI L, RAVAZZOLA M, STORCH MJ, ANDERSON RGW, VASSALLI JD AND PERRELET A. (1987). Proteolytic maturation of insulin is a post-Golgi event which occurs in acidifying clathrin-coated secretory vesicles. *Cell*, **49**, 865–868.

ORCI L, HALBAN P, PERRELET A, AMHERDT M, RAVAZZOLA M AND ANDERSON RGW. (1994). pH-independent and -dependent cleavage of proinsulin in the same secretory vesicle. *J. Cell. Biol.*, **126**, 1149–1156.

PUOPOLO K AND FORGAC M. (1990). Functional reassembly of the coated vesicle proton pump. *J. Biol. Chem.*, **265**, 14836–14841.

SAMBROOK J, FRITSCH EF AND MANIATIS T. (1989). *Molecular Cloning: A Laboratory Manual*. 2nd edn. Cold Spring Harbor Laboratory: New York.

SUN SZ, XIE XS AND STONE DK. (1987). Isolation and reconstitution of the dicyclohexylcarbodiimide-sensitive proton pore of the clathrin-coated vesicle proton translocating complex. *J. Biol. Chem.*, **262**, 14790–14794.

SWALLOW CJ, GRINSTEIN S AND ROTSTEIN O. (1990). A vacuolar type $H^+$-ATPase regulates cytoplasmic pH in murine macrophages. *J. Biol. Chem.*, **265**, 7645–7654.

TAPPER H AND SUNDLER R. (1992). Cytosolic pH regulation in mouse macrophages. *Biochem. J.*, **281**, 245–250.

VAANANEN HK, KARHUKORPI EK, SUNDQUIST K, WALLMARK B, ROININEN I, HENTUNEN T, TUUKKANEN J AND LAKKAKORPI P. (1990). Evidence for the presence of a proton pump of the vacuolar $H^+$-ATPase type in the ruffled borders of osteoclasts. *J. Cell Biol.*, **111**, 1305–1311.

VERLANDER JW, MADSEN KM AND TISHER CC. (1991). Structural and functional features of proton and bicarbonate transport in the rat collecting duct. *Semin. Nephrol.*, **11**, 465–477.

WANG TY AND COX RA. (1968). Isolation of total nucleic acid. In *Methods in Enzymology*. Colowick SP, Kaplan NO (eds) Vol. 12, pp. 115–129, Academic Press: New York.

XIE XS AND STONE DK. (1988). Partial resolution and reconstitution of the subunits of the clathrin-coated vesicle proton ATPase responsible for $Ca^{2+}$-activated ATP hydrolysis. *J. Biol. Chem.*, **263**, 9859–9867.

YAMANAKA Y, FRIESS H, BUCHLER M, BEGER HG, UCHDAA E, ONDA M, KOBRIN MS AND KORC M. (1993). Overexpression of acidic and basic fibroblast growth factors in human pancreatic cancer correlates with advanced tumor stage. *Cancer Res.*, **53**, 5289–5296.

ZHANG J, MEYERS M AND FORGAC M. (1992). Characterization of the $V_0$ domain of the coated vesicle ($H^+$)-ATPase. *J. Biol. Chem.*, **267**, 9773–9778.